# The Association between the Frequency of Rhegmatogenous Retinal Detachment and Atmospheric Temperature

**DOI:** 10.1155/2020/2103743

**Published:** 2020-07-22

**Authors:** Dong Yoon Kim, Hyeseong Hwang, Jae-Hyung Kim, Byung Gil Moon, Sung Min Hyung, Jin Young Kim, Ju Byung Chae

**Affiliations:** ^1^Department of Ophthalmology Chungbuk National University Hospital, College of Medicine, Chungbuk National University, Cheongju, Republic of Korea; ^2^Seoul Daabom Eye Center, Cheongju, Republic of Korea; ^3^Seoul Shinsegae Eye Center, Uijeongbu, Republic of Korea; ^4^Department of Ophthalmology, Jeju National University Hospital, Jeju National University School of Medicine, Jeju, Republic of Korea

## Abstract

Rhegmatogenous retinal detachment (RRD) frequency was observed to be higher with an increase in the daily temperature range. This showed that a wide daily range of temperature, rather than the absolute value of the temperature, is associated with the occurrence of RRD. *Purpose*. To investigate the association between the frequency of rhegmatogenous retinal detachment (RRD) and the atmospheric temperature. *Method*. A retrospective review of consecutive eyes that had undergone primary RRD surgery from 1996 to 2016 at Chungbuk National University Hospital was conducted. Temperature data (highest, lowest, and mean daily temperatures and daily temperature range) in Chungbuk Province were obtained from the Korean Meteorological Administration database. We investigated the relationship between the daily temperature range and the frequency of RRD surgery. We also analyzed the association between various temperature data and the frequency of RRD surgery. *Result*. There were 1,394 RRD surgeries from 1996 to 2016. Among them, 974 eyes were included in this study. The monthly average number of RRD operations showed a bimodal peak (in April and October) throughout the year. With the same tendency as the frequency of RRD, the monthly average of the daily temperature range over 1 year also showed a bimodal peak in April and October. There was a significant positive correlation between the monthly average of the daily temperature range and the number of RRD surgeries (*r* = 0.297, *P* < 0.001). However, there were no associations between RRD frequency and the mean temperature, highest temperature, and lowest temperature. *Conclusion*. The higher the daily temperature range, the higher was the RRD frequency observed. We speculated that dynamic changes in temperature during the day may affect degrees in chorioretinal adhesion and liquefaction of the vitreous, which may eventually result in retinal detachment. Therefore, further experimental studies on the correlation between temperature changes and retinal detachment are needed.

## 1. Introduction

Rhegmatogenous retinal detachment (RRD) is a major cause of blindness and is characterized by the separation of the neurosensory retina from the inner retinal pigment epithelium. The annual incidence of RRD incidence varies according to the time and place (6.9–18.2 per 100,000 persons) [1–7]. In Korea, the average incidence of RRD requiring surgeries was 10.39 cases per 100,000 person-years [8]. The known risk factors for RRD are myopia, long axial length, a history of trauma, congenital anomalies, age, cataract extraction, and RRD in the fellow eye [9–17].

Regarding temperature, there were controversies about the relationship between temperature and RRD incidence. Some authors reported that there was no apparent seasonal variation in the occurrence of new RRD cases [18, 19]. However, several previous studies reported that there was convincing evidence for a seasonal variation of RRD incidence [20–22]. Possible mechanisms of seasonal variation in RRD were as follows: (1) increased temperature associated with vitreous dehydration, (2) low temperatures resulted in increased adhesive forces and protection of the vitreous gel from collapsing, (3) increased physical activities in warmer months, (4) ultraviolet light-mediated increased incidence of posterior vitreous detachment (PVD), and (5) eye rubbing, secondary to allergies [19, 23–25].

Chorioretinal adhesion was an important factor for the normal retinal adhesion and was affected by various factors such as passive hydrostatic forces, interdigitation of outer segments and RPE microvilli, the active transport of subretinal fluid, and metabolic activities [26–28]. Chorioretinal adhesion power also could be affected by temperature change [26, 29–33]. One experimental study reported that the chorioretinal adhesion was increased with low temperature (4°C), compared with that at high temperature (37°C) [31]. Additionally, it was shown that retinal adhesiveness rapidly decreased postmortem at 37°C, but remained near control levels for hours at 4°C [33].

From the point of view that chorioretinal adhesion power was decreased at high temperatures, several reports have published that RRD incidence was increased in the summer. However, there were still controversies about the seasonal variation of RRD incidence. We speculated that the diurnal temperature range, rather than the rise of the temperature, may further affect the occurrence of RRD. We hypothesized that dynamic changes in temperature during the day caused dynamic changes in chorioretinal adhesion and vitreous status, which eventually resulted in the occurrence of retinal detachment. Therefore, we evaluated the association between the frequency of RRD and the daily temperature range.

## 2. Methods

A retrospective review was conducted, of consecutive eyes that had undergone primary RRD surgery (pars plana vitrectomy or scleral buckle) from 1996 to 2016, at Chungbuk National University Hospital. Temperature data (highest, lowest, and mean daily temperatures and daily temperature range) in the Chungbuk province was obtained from the Korean Meteorological Administration database. This study was approved by the Institutional Review Board of Chungbuk National University Hospital and followed the tenets of the Declaration of Helsinki. And because of the retrospective study design, this research involved no more than minimal risk to the subjects. Therefore, the IRB gave exemption of the requirement for obtaining informed consent.

Inclusion criteria included (1) acute onset RRD eyes which had undergone surgical treatment (pars plana vitrectomy or scleral buckle). Exclusion criteria were as follows: (1) chronic RRD (symptom duration ≥ 2 weeks), (2) RRD due to secondary causes such as trauma, inflammation, and surgical treatment, (3) patients with complicated cataract surgery such as vitreous loss, and (4) recurred RRD. The primary objective of this study is to investigate the relationship between the daily temperature range and the frequency of RRD surgery. The secondary objectives of this study are (1) to investigate the relationship between the daily temperature range and the frequency of RRD surgery with respect to age and sex and (2) to analyze the association between various temperature data and the frequency of RRD surgery.

### 2.1. Statistical Analyses

Data are presented as mean ± standard deviation where applicable. Pearson correlation analysis was conducted to evaluate the relationship between RRD frequency and various temperature data (daily temperature range and highest, mean, and lowest temperature). All statistical analyses were performed using SPSS statistical software (version 21.0, SPSS, Inc., Chicago, IL) and statistical significance was defined as *P* < 0.05.

## 3. Results

From 1996 to 2016, there were 1,394 RRD surgeries at Chungbuk National University. Among them, 974 eyes with RRD were included in this study.  Table 1 shows baseline characteristics of the included patients. The participants consisted of 522 men (53.6%) and 452 women (46.4%) with an average age of 49.50 ± 17.59 years. 479 (49.2%) and 415 (42.6%) patients underwent primary vitrectomy and scleral bucking, respectively.

### 3.1. RRD Frequency and Daily Temperature Range

**Figure 1:**
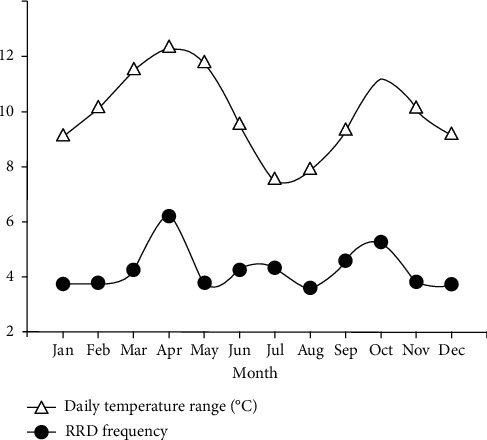
The number of rhegmatogenous retinal detachment (RRD) surgeries and the respective daily temperature ranges. The monthly average number of RRD surgeries showed a bimodal peak (April and October) over the year. With the same tendency as the frequency of RRD, the monthly average of the daily temperature range for 1 year also showed a bimodal peak in April (daily temperature range, 12.3°C) and October (daily temperature range, 11.2°C).

**Figure 2:**
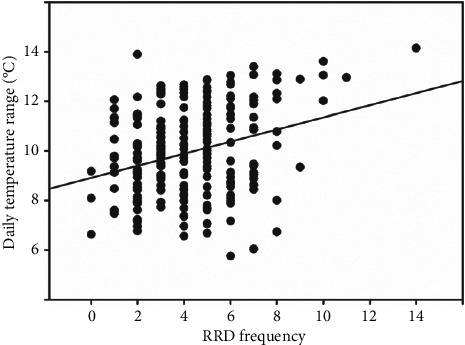
The correlation between rhegmatogenous retinal detachment (RRD) frequency and daily temperature range. There was a significant positive correlation between the monthly average of the daily temperature range and the number of RRD surgeries (Pearson correlation coefficient: 0.297, *P* < 0.001).

Figure 1 shows the numbers of RRD surgeries and the daily temperature range. The monthly average number of RRD operations showed a bimodal peak (April and October) during the year. With the same tendency as the frequency of RRD, the monthly average of the daily temperature range over 1 year also showed a bimodal peak in April (daily temperature range, 12.3°C) and October (daily temperature range, 11.2°C).  Figure 2 shows the correlation between RRD frequency and daily temperature range. A significant positive correlation was found between the monthly average of the daily temperature range and the number of RRD surgeries (Pearson correlation coefficient: 0.297, *P* < 0.001).

### 3.2. RRD Frequency and Daily Temperature Range according to Sex and Age

**Figure 3:**
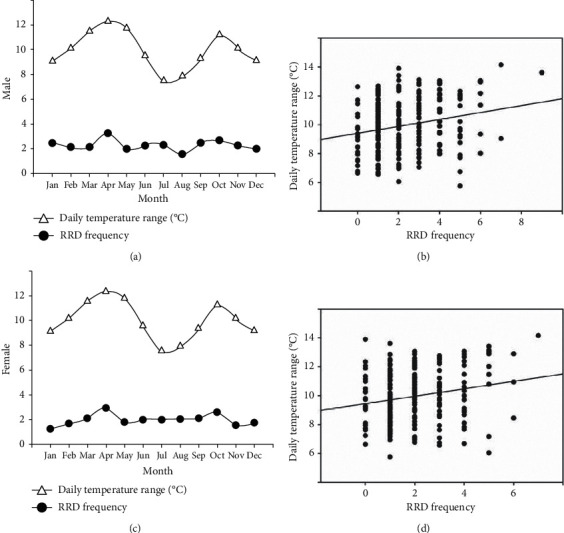
Rhegmatogenous retinal detachment (RRD) frequency and daily temperature range according to sex. Even on separate analysis of men and women, the monthly average number of RRD surgeries showed a bimodal peak (April and October) throughout the year and a significant positive correlation was found between the monthly average of daily temperature range and the number of RRD surgeries (male, Pearson correlation coefficient: 0.220, *P*=0.001; female, Pearson correlation coefficient: 0.203, *P*=0.002).

Even on separate analysis of men and women, the monthly average number of RRD surgeries showed a bimodal peak (April and October) throughout the year and there was a significant positive correlation between the monthly average of the daily temperature range and the number of RRD surgeries (Figure 3).

**Figure 4:**
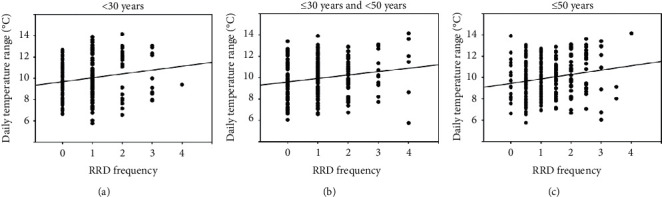
Rhegmatogenous retinal detachment (RRD) frequency and daily temperature range according to age. A positive correlation was found between the monthly average of daily temperature ranges and the number of RRD surgeries, regardless of age (<30 years, Pearson correlation coefficient: 0.174, *P*=0.009; ≤30 years and <50 years, Pearson correlation coefficient: 0.179, *P*=0.007; ≤50 years, Pearson correlation coefficient: 0.192, *P*=0.004).

Figure 4 shows the number of RRD surgeries and the daily temperature range according to age (<30 years, ≤30 years and <50 years, and ≤50 years). A positive correlation was also found between the monthly average of the daily temperature range and the number of RRD surgeries, regardless of the age group.

### 3.3. RRD Frequency and Mean Temperature

**Figure 5:**
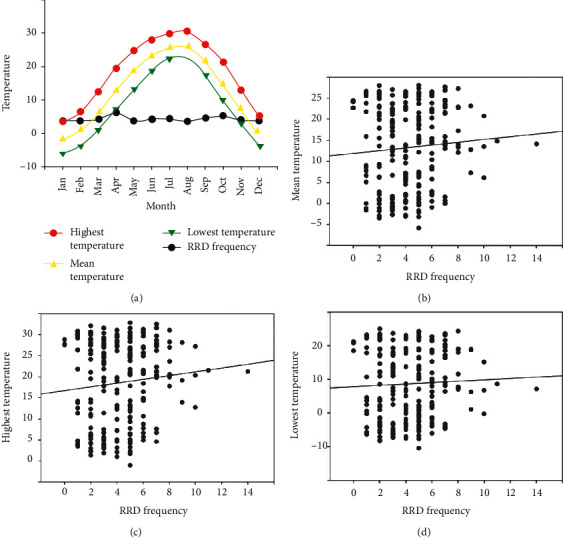
Rhegmatogenous retinal detachment (RRD) frequency and absolute temperature. There was no correlation between RRD frequency and the highest, lowest, and mean temperatures.

We analyzed the relationship between RRD frequency and the highest, lowest, and mean temperatures. Unlike daily temperature ranges, there was no correlation between RRD frequency and the highest, lowest, and mean temperatures (Figure 5).

## 4. Discussion

The primary finding of this study was that both the frequency of RRD and the monthly average of daily temperature ranges showed a bimodal peak in April and October. The higher the daily temperature range, the higher was the RRD frequency observed. However, the highest, lowest, and mean temperatures were not associated with RRD frequency.

The vitreous state such as occurrence of posterior vitreous detachment (PVD) and chorioretinal adhesion power are related to the development of RRD. Myopia, long axial length, a history of trauma, age, and cataract extraction are not only risk factors for RRD, but also contributing factors for the development of PVD [9–14, 34]. PVD itself may cause a retinal tear, which, if left untreated, frequently results in RRD [35, 36]. RRD could also occur when the forces of adhesion between the neurosensory retina and the retinal pigment epithelium are weakened [7]. Therefore, there might be an increased chance to develop RRD, when chorioretinal adhesion is decreased.

The decrease in PVD and chorioretinal adhesion power, which can increase the chance of RRD occurrence, could be affected by temperature [19, 25, 27, 33, 37]. Rahman et al. reported a significant increase in the number of occurrences of PVD with increasing weekly temperatures [38]. In addition to PVD occurrence, the temperature changes could cause contraction of the vitreous cytoskeleton, such as collagen fibrils, which eventually produce traction forces on the retina and RRD [39, 40]. It is known that chorioretinal adhesion power changes according to the temperature. Yao et al. reported that chorioretinal adhesion was increased at 4°C, compared to 37°C [31]. A similar tendency was reported in one postmortem study. Marmor et al. reported that retinal adhesiveness decreased rapidly postmortem at 37°C, but remained near control levels for hours at 4°C [33]. Taken together, we know that at the high temperatures, the occurrence of PVD and chorioretinal adhesion was increased and decreased, respectively. Considering that the development of PVD and chorioretinal adhesion power may be changed according to temperature, temperature can also affect the occurrence of retinal detachment.

There were several reports about the relationship between RRD and temperature. However, the results of previous studies on the relationship between the occurrence of retinal detachment and air temperature were often in conflict with each other. Thelen et al. reported in a large retrospective review of 2,314 patients with primary RRD in Germany a significant midsummer peak incidence (numbers in July = 228) and winter trough (mean numbers from December to January = 161) with a difference of 36% between the two periods [41]. Mansour et al. reported a significant increase in RRD in spring and summer compared to autumn and winter (56% vs. 44%). Interestingly, there was a significantly younger age of onset of RRD in the warmer months (47 vs. 54; *P*=0.007) [20]. On the other hand, Samarrai et al. reported a peak incidence in winter (with the highest level in November) and a trough in the summer months [42]. Some authors reported that there was no apparent seasonal variation in the occurrence of new RRD cases [18, 19]. Li reported a relationship between temperature and RRD incidence with 478 cases. They reported no apparent seasonal variation in the occurrence of new RRD cases during the 1-year period. They also found no apparent seasonal variation when patients were further classified into the three types of RRD (blunt traumatic, aphakic and pseudophakic, and nontraumatic phakic) [18].

Unlike the previous study, which only analyzed the absolute value of temperature, our study focused on the effect of temperature change with respect to RRD. From this study, we could learn that the daily temperature range, rather than the absolute value of the temperature, was associated with the occurrence of RRD. We found that there is a significant correlation between the occurrence of RRD and daily temperature range, and a sudden temperature change compared to the previous month was also related to the occurrence of RD. Due to lack of experimental studies, we can just speculate about the reason why changes in temperature rather than the absolute values of the temperature may affect retinal detachment. A large change in the temperature in the middle of the day causes a large temperature change in the inside of the eyeball. This may lead to dynamic changes in the vitreous (vitreous liquefaction or contraction) and a decrease in the degree of chorioretinal adhesion, ultimately resulting in RRD. In addition, particularly, changes in vitreous state and chorioretinal adhesion due to temperature changes may have a great influence on the patients who have undiagnosed flap retina tear or localized retinal detachment with vitreoretinal traction. We thought that abrupt changes in temperature in these eyes might lead to progression to the surgery requiring RRD. However, to conclude that high daily temperature range can affect the increased incidence of RRD, further basic research must be needed about the correlation between temperature changes and retinal detachment because there is no relevant experimental research.

Though we found a positive correlation between daily temperature range and RRD frequency, such association between diurnal variation and retinal detachment might be caused by other reasons. Other possible reasons for the bimodal monthly incidence of acute rhegmatogenous retinal detachment are as follows: (1) In April and October, the daily temperature range is large, but the overall temperature is good for outdoor activity, thus increasing such activity in April and October. Increased outdoor activity in April and October may have affected the incidence of retinal detachment. (2) Atmospheric pollen level also increases in April and October. An increase in pollen causes allergies (April and October), which is a factor that increases eye rubbing. Since eye rubbing is a major triggering factor for retinal detachment especially in atopic patients, an increase in atmospheric pollen, rather than the daily temperature range, may have affected the occurrence of retinal detachment [43, 44].

This study had several limitations: (1) As we described in the previous paragraph, the association between diurnal variation and retinal detachment could be caused by many other contributing factors, such as increased outdoor activity and pollen at that season. (2) In this study, we could not include horseshoe or flap retinal tear patients without retinal detachment, which might also be associated with PVD. To identify the true effect of temperature on the PVD or chorioretinal adhesion status, they should be included. However, because most horseshoe or flap retinal tear patients without retinal detachment were treated at the primary and secondary ophthalmologic clinic, we could not figure out the exact numbers of retina breaks without retinal detachment patients. (3) Although Chungbuk National University Hospital is the only retina surgical center in the Chungbuk province with a population of 1.5 million, it is not a completely isolated area, so some patients from outside the area may undergo surgery here. Therefore, the number of included patients does not reflect the total number of retinal detachment patients in the Chungbuk province during the study period. (4) We could not analyze RD frequency from different locals with more extreme temperature fluctuations or minimal daily temperature fluctuations. If we could do further analysis of RD frequency from different locals with more extreme temperature fluctuations or minimal daily temperature fluctuations, we could further strengthen the association between RRD frequency and daily temperature range.

Despite these many limitations, it is a long-term, cumulative study of a relatively large number of patients and may be of significance because it is the first study to report the correlation between diurnal temperature variation and the incidence of retinal detachment. In conclusion, the higher the daily temperature range, the more the RRD frequency was observed. From the point that PVD and chorioretinal adhesion might be affected by temperature, we speculated that dynamic changes in temperature during the day may weaken the chorioretinal adhesion and accelerate the liquefaction of the vitreous, which may eventually result in retinal detachment. Therefore, experimental studies on the correlation between the daily temperature range and retinal detachment are needed in the future.

## Figures and Tables

**Table 1:** Clinical characteristics of the included patients.

	Mean (% or SD)
Number of patients	974
Age	49.50 (17.59)
Sex (M/F)	522/452
Right/left	524/450
Operation type	
Primary sclera buckling	479 (49.2%)
Primary vitrectomy	415 (42.6%)
Combined (vitrectomy + sclera buckling)	80 (8.2%)

## Data Availability

The data used to support the findings of this study are restricted by the Institutional Review Board of Chungbuk National University Hospital in order to protect patient privacy. Data are available from Dong Yoon Kim (umlover9@gmail.com) for researchers who meet the criteria for access to confidential data.

## References

[1] Haugstad M., Moosmayer S., Bragadomicronttir R. (2016). Primary rhegmatogenous retinal detachment - surgical methods and anatomical outcome. *Acta Ophthalmologica*.

[2] Poulsen C. D., Peto T., Grauslund J., Green A. (2016). Epidemiologic characteristics of retinal detachment surgery at a specialized unit in Denmark. *Acta Ophthalmologica*.

[3] Chen S.-N., Lian I.-B., Wei Y.-J. (2016). Epidemiology and clinical characteristics of rhegmatogenous retinal detachment in Taiwan. *British Journal of Ophthalmology*.

[4] Törnquist R., Stenkula S., Törnquist P. (1987). Retinal detachment. A study of a population-based patient material in Sweden 1971–1981. I. Epidemiology. *Acta Ophthalmologica*.

[5] Laatikainen L., Tolppanen E. M., Harju H. (1985). Epidemiology of rhegmatogenous retinal detachment in a Finnish population. *Acta Ophthalmologica*.

[6] Wilkes S. R., Beard C. M., Kurland L. T., Robertson D. M., O’Fallon W. M. (1982). The incidence of retinal detachment in Rochester, Minnesota, 1970–1978. *American Journal of Ophthalmology*.

[7] Haimann M. H., Burton T. C., Brown C. K. (1982). Epidemiology of retinal detachment. *Archives of Ophthalmology*.

[8] Park S. J., Choi N. K., Park K. H., Woo S. J. (2013). Five year nationwide incidence of rhegmatogenous retinal detachment requiring surgery in Korea. *PLoS One*.

[9] Hajari J. N., Bjerrum S. S., Christensen U., Kiilgaard J. F., Bek T., la Cour M. (2014). A nationwide study on the incidence of rhegmatogenous retinal detachment in Denmark, with emphasis on the risk of the fellow eye. *Retina*.

[10] Bjerrum S. S., Mikkelsen K. L., La Cour M. (2013). Risk of pseudophakic retinal detachment in 202 226 patients using the fellow nonoperated eye as reference. *Ophthalmology*.

[11] Rowe J. A., Erie J. C., Baratz K. H. (1999). Retinal detachment in olmsted county, Minnesota, 1976 through 1995. *Ophthalmology*.

[12] Johnston P. B. (1991). Traumatic retinal detachment. *British Journal of Ophthalmology*.

[13] Mitry D., Singh J., Yorston D. (2011). The predisposing pathology and clinical characteristics in the Scottish retinal detachment study. *Ophthalmology*.

[14] Cambiaggi A. (1964). Myopia and retinal detachment. *American Journal of Ophthalmology*.

[15] Kim M. S., Park S. J., Park K. H., Woo S. J. (2019). Different mechanistic association of myopia with rhegmatogenous retinal detachment between young and elderly patients. *BioMed Research International*.

[16] Fujiwara N., Tomita G., Yagi F. (2020). Incidence and risk factors of iatrogenic retinal breaks: 20-gauge versus 25-gauge vitrectomy for idiopathic macular hole repair. *American Journal of Ophthalmology*.

[17] Kassem R., Greenwald Y., Achiron A. (2018). Peak occurrence of retinal detachment following cataract surgery: a systematic review and pooled analysis with internal validation. *American Journal of Ophthalmology*.

[18] Li X., Beijing G. (2003). Rhegmatogenous retinal detachment study. Incidence and epidemiological characteristics of rhegmatogenous retinal detachment in beijing, China. *Ophthalmology*.

[19] Ivanisevic M., Erceg M., Eterovic D. (2002). Rhegmatogenous retinal detachment and seasonal variations. *Acta Med Croatica*.

[20] Mansour A. M., Hamam R. N., Sibai T. A., Farah T. I., Mehio-Sibai A., Kanaan M. (2009). Seasonal variation of retinal detachment in Lebanon. *Ophthalmic Research*.

[21] Bertelmann T., Cronauer M., Stoffelns B., Sekundo W. (2011). Saisonale Variation des Auftretens rhegmatogener Netzhautablösungen zu Beginn des 21. Jahrhunderts. *Der Ophthalmologe*.

[22] Lin H.-C., Chen C.-S., Keller J. J., Ho J.-D., Lin C.-C., Hu C.-C. (2011). Seasonality of retinal detachment incidence and its associations with climate: an 11-year nationwide population-based study. *Chronobiology International*.

[23] Jensen P. (1957). Seasonal variations in detachment of the retina. *Acta Ophthalmologica*.

[24] Paavola M., Chehova S., Forsius H. (1983). Seasonal variations in retinal detachment in Northern Finland and Novosibirsk. *Acta Ophthalmologica*.

[25] Ghisolfi A., Vandelli G., Marcoli F. (1986). Seasonal variations in rhegmatogenous retinal detachment as related to meteorological factors. *Ophthalmologica*.

[26] Marmor M. F., Abdul-Rahim A. S., Cohen D. S. (1980). The effect of metabolic inhibitors on retinal adhesion and subretinal fluid resorption. *Investigative Ophthalmology & Visual Science*.

[27] Yoon Y. H., Marmor M. F. (1988). Rapid enhancement of retinal adhesion by laser photocoagulation. *Ophthalmology*.

[28] Fatt I., Shantinath K. (1971). Flow conductivity of retina and its role in retinal adhesion. *Experimental Eye Research*.

[29] Marmor M. F., Yao X. Y. (1989). The enhancement of retinal adhesiveness by ouabain appears to involve cellular edema. *Investigative Ophthalmology and Visual Science*.

[30] Endo E. G., Yao X. Y., Marmor M. F. (1988). Pigment adherence as a measure of retinal adhesion: dependence on temperature. *Investigative Ophthalmology & Visual Science*.

[31] Yao X. Y., Endo E. G., Marmor M. F. (1989). Reversibility of retinal adhesion in the rabbit. *Investigative Ophthalmology & Visual Science*.

[32] Yao X. Y., Hageman G. S., Marmor M. F. (1994). Retinal adhesiveness in the monkey. *Investigative Ophthalmology & Visual Science*.

[33] Marmor M. F., Yao X.-Y., Hageman G. S. (1994). Retinal adhesiveness in surgically enucleated human eyes. *Retina*.

[34] Foos R. Y., Wheeler N. C. (1982). Vitreoretinal juncture. *Ophthalmology*.

[35] Johnson M. W. (2010). Posterior vitreous detachment: evolution and complications of its early stages. *American Journal of Ophthalmology*.

[36] Yannuzzi N. A., Chang J. S., Brown G. C., Smiddy W. E. (2018). Cost-utility of evaluation for posterior vitreous detachment and prophylaxis of retinal detachment. *Ophthalmology*.

[37] Yoon Y. H., Marmor M. F. (1988). Effects of retinal adhesion of temperature, cyclic AMP, cytochalasin, and enzymes. *Investigative Ophthalmology &amp; Visual Science*.

[38] Rahman R., Ikram K., Rosen P. H., Cortina-Borja M., Taylor M. E. (2002). Do climatic variables influence the development of posterior vitreous detachment?. *British Journal of Ophthalmology*.

[39] Auger N., Rhéaume M.-A., Bilodeau-Bertrand M., Tang T., Kosatsky T. (2017). Climate and the eye: case-crossover analysis of retinal detachment after exposure to ambient heat. *Environmental Research*.

[40] Stocks S. Z., Taylor S. M., Shiels I. A. (2001). Transforming growth factor-beta1 induces alpha-smooth muscle actin expression and fibronectin synthesis in cultured human retinal pigment epithelial cells. *Clinical and Experimental Ophthalmology*.

[41] Thelen U., Gerding H., Clemens S. (1997). Rhegmatogene netzhautablösungen. *Der Ophthalmologe*.

[42] Al Samarrai R. (1990). Seasonal variations of retinal detachment among Arabs in Kuwait. *Ophthalmic Research*.

[43] Hida T., Tano Y., Okinami S., Ogino N., Inoue M. (2000). Multicenter retrospective study of retinal detachment associated with atopic dermatitis. *Japanese Journal of Ophthalmology*.

[44] Yoneda K., Okamoto H., Wada Y. (1995). Atopic retinal detachment. Report of four cases and a review of the literature. *British Journal of Dermatology*.

